# In vivo analysis of the effect of panobinostat on cell-associated HIV RNA and DNA levels and latent HIV infection

**DOI:** 10.1186/s12977-016-0268-7

**Published:** 2016-05-21

**Authors:** Perry Tsai, Guoxin Wu, Caroline E. Baker, William O. Thayer, Rae Ann Spagnuolo, Rosa Sanchez, Stephanie Barrett, Bonnie Howell, David Margolis, Daria J. Hazuda, Nancie M. Archin, J. Victor Garcia

**Affiliations:** Division of Infectious Diseases, Center for AIDS Research, University of North Carolina at Chapel Hill School of Medicine, 120 Mason Farm Rd., CB 7042, Genetic Medicine Building 2043, Chapel Hill, NC 27599 USA; Merck Research Laboratories, Merck & Co., Inc., West Point, PA 19486 USA

**Keywords:** HIV, Latency, Panobinostat, Histone acetylation, BLT, Humanized mice

## Abstract

**Background:**

The latent reservoir in resting CD4^+^ T cells presents a major barrier to HIV cure. Latency-reversing agents are therefore being developed with the ultimate goal of disrupting the latent state, resulting in induction of HIV expression and clearance of infected cells. Histone deacetylase inhibitors (HDACi) have received a significant amount of attention for their potential as latency-reversing agents.

**Results:**

Here, we have investigated the in vitro and systemic in vivo effect of panobinostat, a clinically relevant HDACi, on HIV latency. We showed that panobinostat induces histone acetylation in human PBMCs. Further, we showed that panobinostat induced HIV RNA expression and allowed the outgrowth of replication-competent virus ex vivo from resting CD4^+^ T cells of HIV-infected patients on suppressive antiretroviral therapy (ART). Next, we demonstrated that panobinostat induced systemic histone acetylation in vivo in the tissues of BLT humanized mice. Finally, in HIV-infected, ART-suppressed BLT mice, we evaluated the effect of panobinostat on systemic cell-associated HIV RNA and DNA levels and the total frequency of latently infected resting CD4^+^ T cells. Our data indicate that panobinostat treatment resulted in systemic increases in cellular levels of histone acetylation, a key biomarker for in vivo activity. However, panobinostat did not affect the levels of cell-associated HIV RNA, HIV DNA, or latently infected resting CD4^+^ T cells.

**Conclusion:**

We have demonstrated robust levels of systemic histone acetylation after panobinostat treatment of BLT humanized mice; and we did not observe a detectable change in the levels of cell-associated HIV RNA, HIV DNA, or latently infected resting CD4^+^ T cells in HIV-infected, ART-suppressed BLT mice. These results are consistent with the modest effects noted in vitro and suggest that combination therapies may be necessary to reverse latency and enable clearance. Animal models will contribute to the progress towards an HIV cure.

## Background

Antiretroviral therapy (ART) is able to suppress plasma viral load in HIV-infected patients to undetectable levels, resulting in a reduction in morbidity and mortality. However, these drugs are not able to cure HIV infection, so most patients must remain on treatment indefinitely. The major barrier to cure is that persistent viral infection leads to rebound after ART is interrupted [1]. In addition to ongoing replication in lymphoid tissue sanctuary sites [2], this persistent infection resides as integrated and transcriptionally silent provirus in the genomes of resting CD4^+^ T cells [3–8], creating a latent reservoir defined as a “reversibly nonproductive state of infection” with the “capacity to produce infectious virus particles” [9].

One strategy to cure HIV is to purge the latent reservoir through latency reversal followed by the clearance of infected cells [10–12]. Specifically, latency-reversing agents (LRAs) would induce HIV RNA transcription and viral protein synthesis, followed by death of infected cells mediated by viral cytopathic effects, the host immune system, or a targeted cytotoxic agent. HIV latency is maintained partly by the action of histone deacetylase (HDAC) enzymes. Specifically, deacetylation of histones contributes to a restricted chromatin environment and to transcriptional repression of HIV [13, 14]. To reverse this mechanism, HDAC inhibitors (HDACi) have been extensively investigated as potential LRAs [15–19].

In particular, panobinostat (pan-HDAC inhibitor, LBH589) has been studied for its ability to disrupt HIV latency. Previous studies have shown that panobinostat induces HIV expression in latently infected cell line models like U1 and ACH2 [20], in primary CD4^+^ T cell models of latency [20, 21], and in resting CD4^+^ T cells isolated from chronically infected, ART-suppressed patients [22, 23]. Clinical trials are currently underway in humans [24, 25]; and initial results indicate that, although in vivo administration of panobinostat resulted in a modest (3.5-fold) increase of cell-associated HIV RNA in peripheral blood, it did not reduce the size of the latent reservoir in the panobinostat-treated patients [24].

While HIV cure interventions will ultimately need to be proven effective in humans, animal models such as rhesus macaques or humanized mice are advantageous for preclinical investigation of candidate latency-reversing agents, particularly because of the opportunity to analyze multiple tissue samples other than peripheral blood. A study in SIVmac239-infected ART-suppressed Indian rhesus macaques showed increases in peripheral blood cell-associated HIV RNA after administration of another HDAC inhibitor vorinostat [26]. In contrast, vorinostat did not significantly change viral RNA levels in another study of SIVmac251-infected ART-suppressed Chinese rhesus macaques [27].

Here, we used bone marrow–liver–thymus (BLT) humanized mice to investigate the systemic in vivo effect of panobinostat on histone acetylation, cell-associated HIV RNA, HIV DNA, and latently infected resting CD4^+^ T cells. BLT mice have been shown to recapitulate key features of HIV transmission, infection, pathogenesis, and treatment [28–40]. Furthermore, the frequency of latently infected resting CD4^+^ T cells in HIV-infected, ART-suppressed BLT mice has been previously measured using a quantitative viral outgrowth assay (QVOA) [41]. In this current study, we demonstrated that, despite robust levels of systemic histone acetylation after panobinostat treatment, there were no detectable changes in the levels of cell-associated HIV RNA, HIV DNA, or latently infected resting CD4^+^ T cells. These results are largely consistent with those previously reported in human studies.

## Methods

### Ethics statement

All animal experiments were conducted following guidelines for housing and care of laboratory animals in accordance with University of North Carolina at Chapel Hill (UNC Chapel Hill) regulations after review and approval by the UNC Chapel Hill Institutional Animal Care and Use Committee (permit number 12-171). HIV-infected patients receiving stable, standard-of-care ART with plasma HIV-1 RNA <50 copies/ml and a CD4 count of >300/µl for at least 6 months were enrolled following informed consent. Studies were approved by the UNC Chapel Hill institutional biomedical review board and the Food and Drug Administration.

### Isolation of resting human CD4^+^ T cells for RNA induction and quantitative viral outgrowth assay

Mononuclear cells were isolated from patient leukapheresis products or were pooled from the peripheral blood, lymph nodes, bone marrow, spleen, liver, lung, and thymic organoid of each mouse (one mouse = one pooled sample). Samples from humanized mice were first enriched for human cells using an EasySep Mouse/Human Chimera Isolation Kit (#19849, Stemcell Technologies, Vancouver, Canada). Resting human CD4^+^ T cells were negatively selected by magnetic separation from each sample essentially as described from human leukapheresis product [42] and from BLT mice [41]. Briefly, cells were incubated with antibodies against murine CD45 and TER119 and against human CD8, CD14, CD16, CD19, CD56, glycophorin A, CD41, HLA-DR, CD25 (35 µl/ml) (Stemcell Technologies, Vancouver, Canada), CD31, and CD105 (0.5 µg/ml) (eBiosciences, San Diego, CA). Cells bound to antibody were removed by magnetic separation with EasySep (mice) or StemSep (human) custom isolation kit (Stemcell Technologies, Vancouver, Canada), and the flowthrough containing purified resting human CD4^+^ T cells was collected.

### Measurement of RNA induction and quantitative viral outgrowth from resting cells

10–12 × 10^6^ purified resting CD4^+^ T cells were cultured in media alone (untreated) or panobinostat (20 nM) overnight (18–20 h) for RNA induction, then aliquoted into wells containing 1 × 10^6^ cells each. Total RNA was isolated from each well using the Magmax 96 Total RNA isolation kit (Ambion, Austin, TX). Duplicate pools of cDNA were synthesized from DNase-treated, isolated RNA using the SuperScript III First-Strand Synthesis SuperMix kit (Invitrogen, Carlsbad, CA). Two additional duplicate wells from each treatment condition did not include reverse transcriptase and served as control for DNA contamination. PCR amplification of cDNA was performed in triplicate using the Biorad CFX 96 Real-Time PCR detection system (Biorad, Hercules, CA) with previously published primers and probe [43]. A standard curve was generated for each PCR reaction using cDNA synthesized from in vitro-transcribed RNA where the p5′ plasmid served as template [44]. Results of the triplicate PCR replicates were averaged.

Purified resting cells were maintained in the presence of 15 nM efavirenz or 4 µM abacavir, and 1 µM raltegravir, for 24 h prior to stimulation in limiting dilution with DMSO (0.0002 %, untreated), panobinostat (20 nM), or phytohemagglutinin (PHA, 1 µg/ml) for QVOA [42]. The frequency of infectious units per million or per billion resting human CD4^+^ T cells was calculated as a maximum likelihood estimate (or median posterior estimate if all wells were negative) using an in-house IUPM calculator and IUPMStats v.1.0 [45].

### Generation of BLT humanized mice

BLT mice were generated as described previously [46]. 6- to 8-week-old NSG (NOD.Cg-*Prkdc*^*scid*^*Il2rg*^*tm1Wjl*^/SzJ, stock #5557, The Jackson Laboratory, Bar Harbor, ME) mice were sublethally irradiated, implanted with human thymus and liver tissue, and transplanted with autologous human liver CD34^+^ cells (Advanced Bioscience Resources, Alameda, CA); then they were monitored for human reconstitution in peripheral blood by flow cytometry [41, 46, 47]. All BLT mice (n = 21) used for these experiments contained an average of 51.6 % ± 17.7 SD human CD45^+^ cells in the peripheral blood, of which 60.3 % ± 25.1 SD expressed CD3 on their cell surface. 74.7 % ± 9.3 SD of human CD3^+^ cells expressed human CD4.

### Analysis of histone acetylation

After uninfected human PBMCs were exposed in vitro for 6 h to panobinostat, H3 acetylation was measured by flow cytometry as previously described [48].

H4 acetylation in cells from BLT tissues was assessed by ELISA. Mononuclear cells from each tissue were isolated as previously described [46, 47]. Total cell numbers harvested from each tissue are summarized as follows: average 1.4 × 10^7^ ± 5.3 × 10^6^ SD bone marrow, 1.5 × 10^7^ ± 5.9 × 10^6^ SD liver, 4.3 × 10^6^ ± 2.0 × 10^6^ SD lung, 1.5 × 10^6^ ± 1.1 × 10^6^ SD lymph node, 1.4 × 10^7^ ± 7.3 × 10^6^ SD spleen, 9.7 × 10^7^ ± 1.0 × 10^8^ SD thymic organoid. Cell aliquots were pelleted and resuspended in 200 µl 1 % Triton X-100 (#X100, Sigma-Aldrich, St. Louis, MO) in phosphate-buffered saline (PBS), then further diluted for measurement using 3 % bovine serum albumin (BSA) in PBS. ELISA plates (Corning Costar, Corning, NY) were coated with 2 µg/ml of anti-H4 monoclonal antibody (MBL, Woburn, MA) in 100 µl coating buffer (Sigma-Aldrich, St. Louis, MO) and incubated overnight at 4 °C. Plates were washed for 5 min in 0.05 % Tween 20 (Sigma-Aldrich, St. Louis, MO) in PBS followed by blocking with 3 % BSA/PBS for 2 h to minimize non-specific binding. 100 µl cell lysates were added along with 50 µl 1:500 anti-H4K5/8/12/16 monoclonal antibody (Millipore, Billerica, MA) conjugated to alkaline phosphatase. Plates were incubated overnight at 4 °C with gentle shaking followed by five washes with 0.05 % Tween 20/PBS and gentle shaking. 100 µl of Tropix CDP-Star Sapphire II substrate (Applied Biosystems, Carlsbad, CA) was added to each well, and plates were incubated at room temperature for 20 min, followed by measurement of luminescence counts using an EnVision plate reader (Perkin Elmer, Waltham, MA).

### HIV infection and treatment of BLT mice

BLT mice were infected by intravenous exposure to 3 × 10^4^ tissue culture infectious units HIV-1_JR-CSF_. Infection was monitored in peripheral blood by measuring plasma levels of HIV RNA as previously described (limit of detection = 750 copies per ml from 40 µl plasma sample volume) using one-step reverse transcriptase real-time PCR (custom TaqMan Assays-by-Design, Applied Biosystems, Grand Island, NY) [32, 41]. Tissues were harvested and cells isolated as previously described [46, 47]. Total cell numbers harvested from each tissue are summarized as follows: average 9.2 × 10^7^ ± 3.1 × 10^7^ SD bone marrow, 1.6 × 10^7^ ± 7.6 × 10^6^ SD liver, 5.9 × 10^6^ ± 4.1 × 10^6^ SD lung, 1.3 × 10^6^ ± 1.6 × 10^6^ SD lymph node, 1.5 × 10^6^ ± 1.3 × 10^6^ SD peripheral blood, 1.8 × 10^7^ ± 2.1 × 10^7^ SD spleen, 1.0 × 10^8^ ± 9.5 × 10^7^ SD thymic organoid. Cells were aliquoted for HIV DNA quantification (limit of detection = 4.5 copies), HIV RNA quantification (limit of detection = 4.5 copies), and flow cytometric analysis [46, 47]. Due to the use of carrier RNA during RNA extraction, HIV RNA measurements were normalized to the number of human CD4^+^ T cells. Flow cytometry data were collected using a BD FACSCanto cytometer and analyzed using BD FACSDiva software v. 6.1.3 (BD Biosciences, San Jose, CA).

Antiretroviral therapy was administered to BLT mice via 1/2″ pellets of irradiated Teklad chow 2020X containing 1500 mg emtricitabine, 1,560 mg tenofovir disoproxil fumarate, and 600 mg raltegravir per kg (Research Diets, New Brunswick, NJ). Panobinostat (LBH589, #S1030, Selleckchem, Houston, TX) was dissolved in DMSO then diluted in 10 % (2-hydroxypropyl)-beta-cyclodextrin (#H107, Sigma-Aldrich, St. Louis, MO) (0.4 % DMSO final concentration) for intraperitoneal administration at a dose of 2 mg/kg. This dose was chosen after a higher dose of 5 mg/kg over 2 weeks (four doses, 3–4 days apart) resulted in 60 % (3/5) mortality.

### Statistical tests

**Fig. 1:**
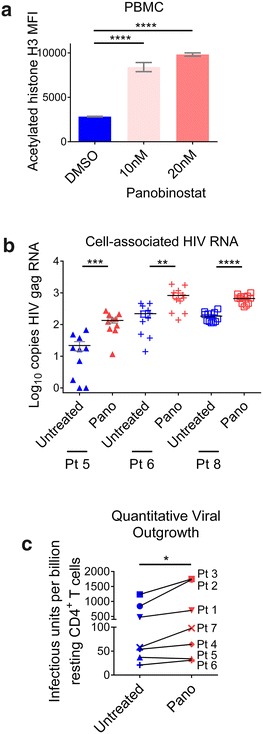
Effect of panobinostat on histone acetylation, HIV RNA, and viral outgrowth from patient cells. **a** Human PBMCs (n = 3) were incubated with panobinostat or DMSO control for 6 h, and histone H3 acetylation was measured by flow cytometry. **b** Resting CD4^+^ T cells were isolated from leukapheresis product obtained from three HIV-infected patients on suppressive antiretroviral therapy (outlined in Table 1), then pulsed with panobinostat (20 nM) or untreated. HIV RNA levels were measured from 10 to 12 individual wells (1 × 10^6^ cells each) by quantitative real-time PCR. **c** Resting CD4^+^ T cells were isolated from leukapheresis product obtained from seven HIV-infected patients on suppressive antiretroviral therapy (outlined in Table 1), then incubated with panobinostat (20 nM) or untreated. Viral outgrowth was measured by QVOA. Mean and SEM plotted with comparison by unpaired t test in **a**; Mann–Whitney test in **b**; Wilcoxon matched-pairs signed rank test in **c**: ns p > 0.05, *p < 0.05, **p < 0.01, ***p < 0.001, ****p < 0.0001. *Blue bars*/*symbols* denote control; *pink* and *red bars*/*symbols* denote panobinostat-treated

**Fig. 2:**
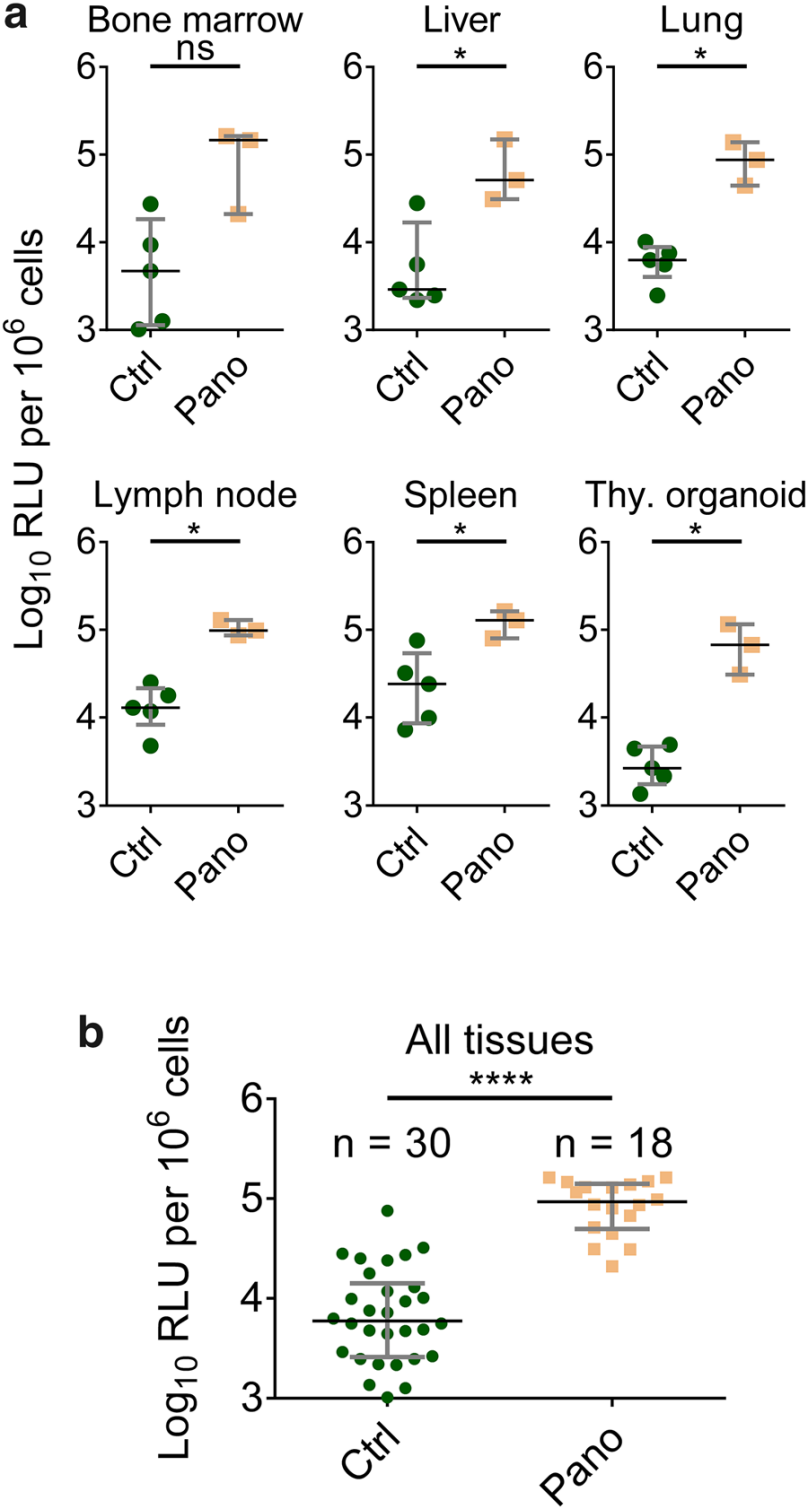
Panobinostat administration induces systemic histone acetylation. NSG/BLT mice were administered panobinostat (2 mg/kg, n = 3) or vehicle (n = 5) intraperitoneally. After 24 h, tissues were harvested, and cells were isolated and resuspended in 1 % Triton-X/PBS. Cell lysates were analyzed for histone acetylation by ELISA. **a** Data from individual tissues. **b** Data from all tissues. Median and interquartile range plotted with comparisons by Mann–Whitney test: ns p > 0.05, *p < 0.05, ****p < 0.0001. *Green circles* denote control; *mustard squares* denote panobinostat-treated

All statistical tests were performed using an alpha level of 0.05. Unpaired t test was utilized in Fig. 1a. Mann–Whitney test was utilized in Figs. 1b, 2, 4, 5 and 6. Wilcoxon matched-pairs signed rank test was utilized in Fig. 1c. Graphs were generated in Graphpad Prism (v. 6).

## Results

### Induction of HIV expression with panobinostat from resting CD4^+^ T cells isolated from HIV-infected patients on suppressive antiretroviral therapy

First, we assessed the effect of panobinostat on histone H3 acetylation in uninfected human PBMCs by flow cytometry. After 6 h incubation with panobinostat, there was a 3.0-fold increase in the level (mean fluorescence intensity) of histone acetylation in PBMCs with 10 nM panobinostat relative to DMSO control and a 3.5-fold increase with 20 nM panobinostat (p < 0.0001) (Fig. 1a).

In order to measure the effect of panobinostat on latently infected cells, we isolated resting CD4^+^ T cells from HIV-infected patients who were durably suppressed on ART for at least 6 months (Table 1). We incubated resting cells from three patients with 20 nM panobinostat overnight and measured levels of cell-associated HIV RNA. The levels of HIV RNA increased by 6.2-fold (p < 0.001), 3.7-fold (p < 0.01), and 3.6-fold (p < 0.0001) in cells from patients 5, 6, and 8, respectively, in comparison to untreated cells (Fig. 1b). Resting CD4^+^ T cells from seven patients were also plated by limiting dilution and incubated with or without panobinostat (20 nM) to determine quantitative viral outgrowth. The mean infectious units per billion cells detected with untreated cells was 389, and the mean with panobinostat-treated cells was 630 (p < 0.05) (Fig. 1c). Together, these results demonstrate that panobinostat induced histone acetylation in primary human lymphocytes and allowed the recovery of HIV transcription and viral outgrowth from the resting CD4^+^ T cells of several HIV-infected aviremic patients. Based on these encouraging in vitro results, we proceeded to evaluate the effect of panobinostat in vivo.

**Table 1:** Patient characteristics

Patient	Age	Sex	Race	Time on ART (months)	Time undetectable (months)	Current viral load (copies/ml)	Current CD4 count (cells/µl)
1	27	Male	White	74	70	<40	977
2	48	Male	White	76	>6	<50	720
3	N/A	Male	White	38	32	<40	1145
4	57	Male	White	121	83	<40	487
5	27	Male	Black/African American	53	52	<40	798
6	61	Male	Black/African American	235	110	<20	1302
7	53	Male	White	193	76	<40	746
8	52	Male	White	267	75	<40	439

### In vivo histone acetylation in tissues after treatment with panobinostat

Having characterized the ex vivo activity of panobinostat in primary human cells, we proceeded to perform a systemic in vivo evaluation using BLT humanized mice. BLT mice were administered either vehicle (n = 5) or a single 2 mg/kg dose of panobinostat (n = 3) by intraperitoneal injection. After 24 h, tissues were harvested, and cells were isolated for determination of histone acetylation by ELISA. The levels of histone acetylation were significantly higher in five of the six tissues analyzed (p < 0.05 in liver, lung, lymph node, spleen, and thymic organoid); the difference was also higher in the bone marrow of panobinostat-treated mice with a trend toward significance (p = 0.07) (Fig. 2a). Taken together, a single 2 mg/kg dose of panobinostat resulted in increased histone acetylation 24 h later in all tissues analyzed, with a median RLU of 92,806 per million cells versus 5946 in the vehicle group, a 15.6-fold difference that was highly statistically significant (p < 0.0001) (Fig. 2b). These results indicate that the administration of panobinostat resulted in systemic histone acetylation in vivo in the tissues of BLT mice at this dose and timepoint, and that the determination of levels of histone acetylation can be used as a biomarker for histone deacetylase inhibition in vivo.

### Analysis of the effect of panobinostat treatment in HIV-infected, ART-suppressed BLT mice

**Fig. 3:**
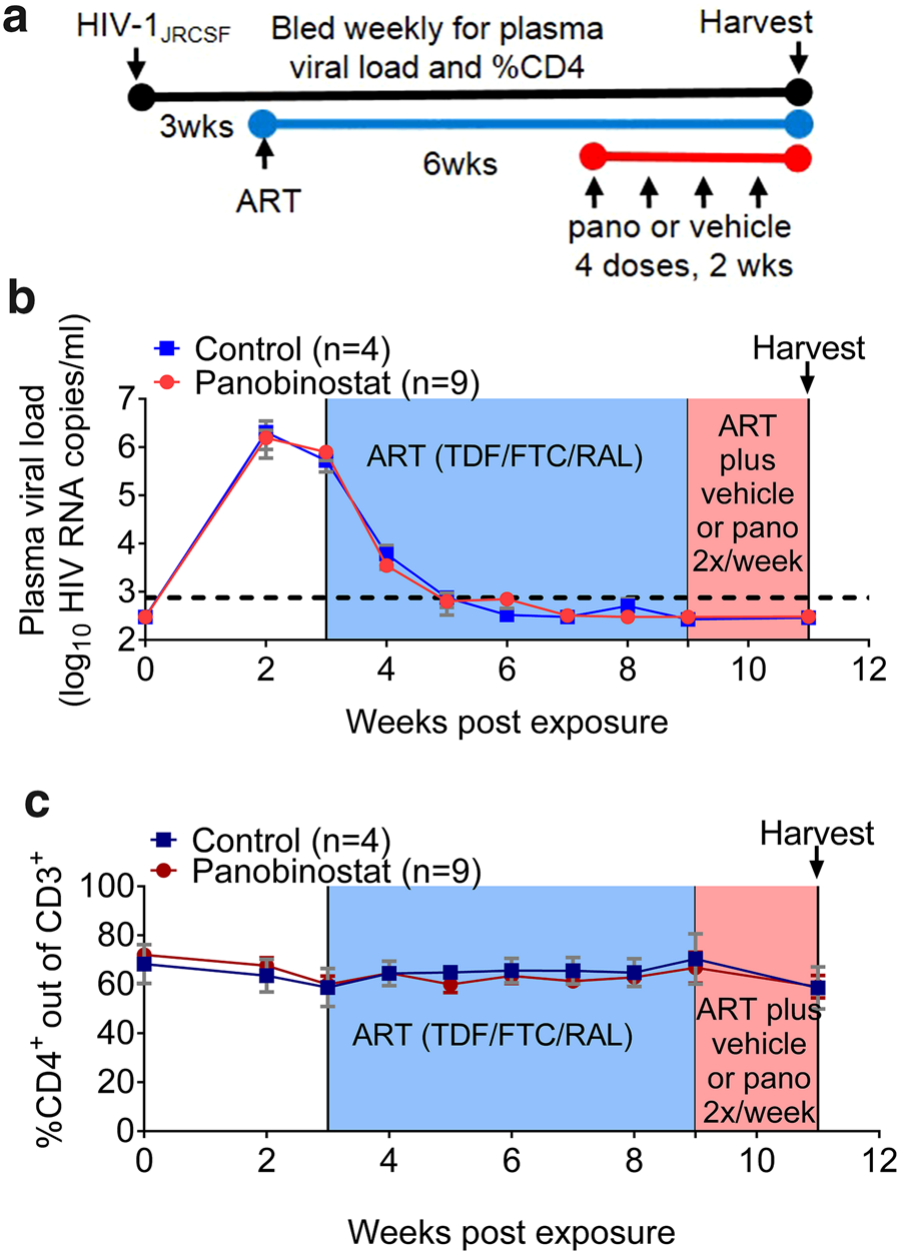
Outline of panobinostat treatment of HIV-infected, ART-suppressed BLT mice. **a** Experimental outline. BLT mice were infected intravenously with HIV-1_JR-CSF_. Starting at 2 weeks post exposure, mice were bled weekly for plasma viral load and flow cytometry analysis. Starting at 3 weeks post exposure, mice were administered antiretroviral therapy (ART) consisting of tenofovir disoproxil fumarate, emtricitabine, and raltegravir (*light blue shading*). After 6 weeks of ART, one group of mice was administered panobinostat at a dose of 2 mg/kg intraperitoneally (*red circles*, n = 9) or vehicle (*blue squares*, n = 4), twice a week for 2 weeks, in addition to ART (*light red shading*). At the end of the experiment, mice were harvested, and cells were isolated for real-time PCR analysis and flow cytometry analysis. **b** Plasma viral load was measured by quantitative real-time PCR (limit of detection = 750 copies/ml, *dashed line*), and **c**  %CD4^+^ T cells was measured by flow cytometry. Mean values and SEM plotted

**Fig. 4:**
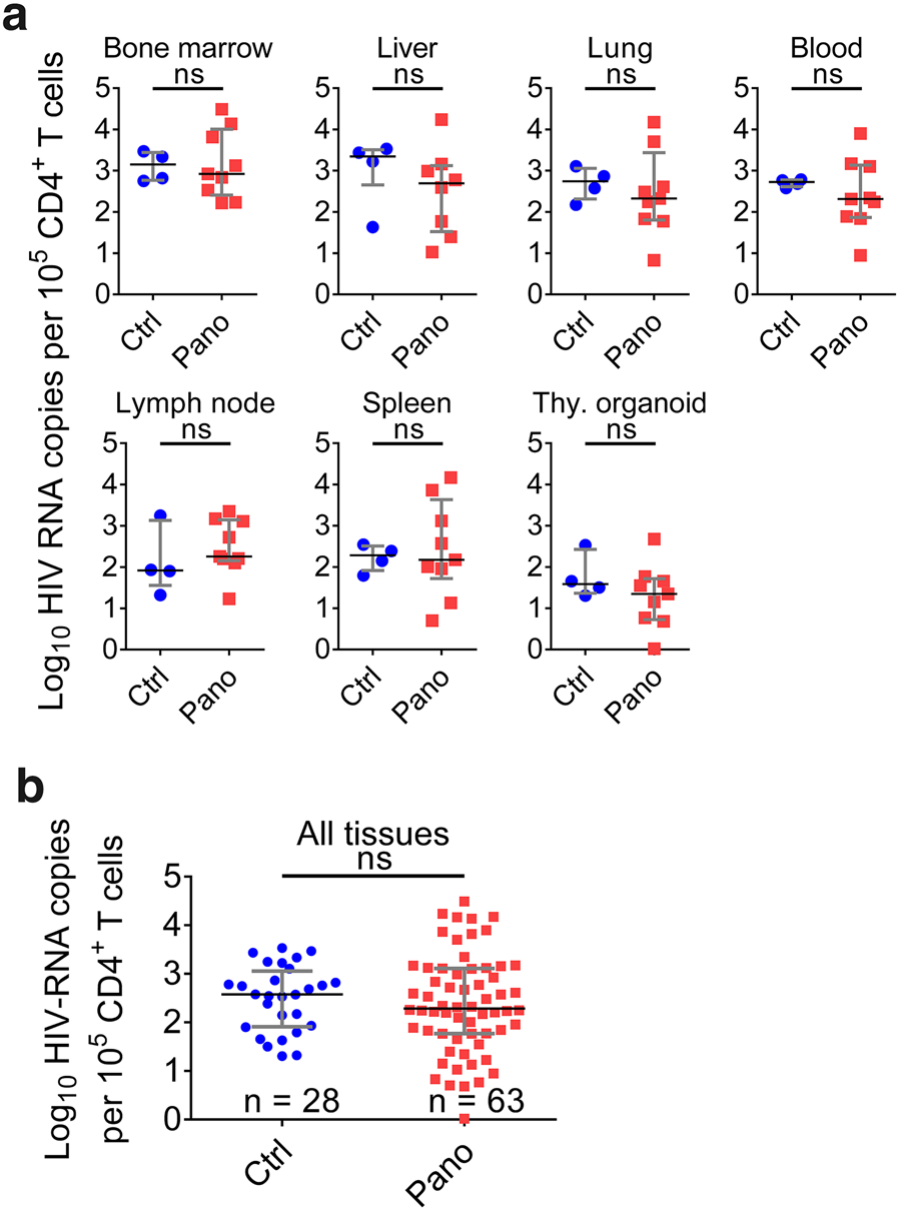
Analysis of cell-associated HIV RNA levels in the tissues of infected, suppressed, panobinostat-treated BLT mice. BLT mice were infected with HIV-1_JR-CSF_, suppressed by antiretroviral therapy, and treated with panobinostat as described in Fig. 3. Cells were then isolated from peripheral blood, bone marrow, liver, lung, lymph nodes, spleen, and thymic organoid for real-time quantitative PCR analysis of cell-associated HIV RNA levels. **a** Data from individual tissues. **b** Data from all tissues. Median and interquartile range plotted with comparisons by Mann–Whitney test: ns p > 0.05. *Blue circles* denote control; *red squares* denote panobinostat-treated

**Fig. 5:**
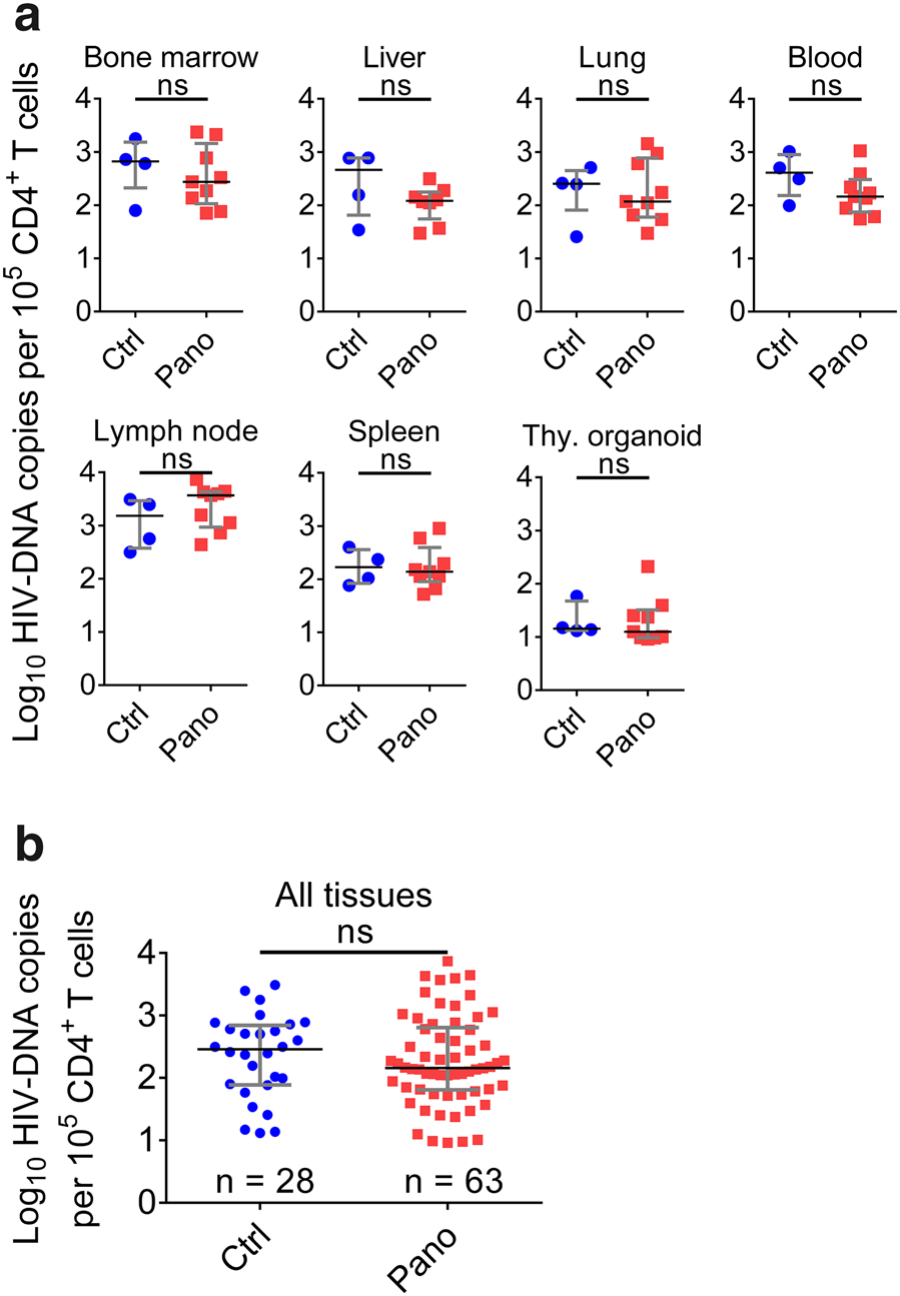
Analysis of HIV DNA levels in the tissues of infected, suppressed, panobinostat-treated BLT mice. BLT mice were infected with HIV-1_JR-CSF_, suppressed by antiretroviral therapy, and treated with panobinostat as described in Fig. 3. Cells were then isolated from peripheral blood, bone marrow, liver, lung, lymph nodes, spleen, and thymic organoid for real-time quantitative PCR analysis of HIV DNA levels. **a** Data from individual tissues. **b** Data from all tissues. Median and interquartile range plotted with comparisons by Mann–Whitney test: ns p > 0.05. *Blue circles* denote control; *red squares* denote panobinostat-treated

In order to evaluate the in vivo effect of panobinostat on cell-associated HIV RNA, BLT mice (n = 13) were infected with HIV-1_JR-CSF_ (3 × 10^4^ tissue culture infectious units) administered once via intravenous inoculation (Fig. 3a). Three weeks after infection all animals had plasma viral loads greater than 1 × 10^6^ HIV RNA copies/ml (Fig. 3b). All infected mice were then administered ART consisting of raltegravir, emtricitabine, and tenofovir as indicated in the Methods section. As early as 2 weeks after initiation of ART, plasma viral loads were below the limit of detection (750 copies/ml) (Fig. 3b) and remained undetectable for the duration of the experiment. Six weeks after therapy initiation, suppressed mice were treated twice a week for 2 weeks with vehicle (n = 4) or panobinostat (2 mg/kg intraperitoneally, n = 9) (total four doses 3–4 days apart), in addition to the ART. At the end of 2 weeks (4 days after last panobinostat administration), tissues from control and panobinostat-treated mice were harvested. Single cell suspensions were prepared from bone marrow, liver, lung, lymph node, spleen, thymic organoid, and peripheral blood, then analyzed by flow cytometry or used to isolate nucleic acids to quantitate HIV RNA and DNA levels by real-time PCR. There were no significant differences (p > 0.05) in the median levels of cell-associated HIV RNA with regard to individual tissues between the control group and panobinostat group (Fig. 4a). With all tissue data points taken together, the median level of cell-associated HIV RNA in the control group was 377 copies per 100,000 CD4^+^ T cells, compared to 193 copies per 100,000 CD4^+^ T cells in the panobinostat group. However, this difference was not statistically significant (p > 0.05) (Fig. 4b). Similar to the RNA results, there were no significant differences in median HIV DNA in individual tissues (Fig. 5a). The median copies HIV DNA per 100,000 CD4^+^ T cells of all tissues analyzed were 287 in the control group and 145 in the panobinostat group, but this difference was not statistically significant (p > 0.05) (Fig. 5b).

### Analysis of the effect of panobinostat on the levels of latently infected resting human CD4^+^ T cells

**Fig. 6:**
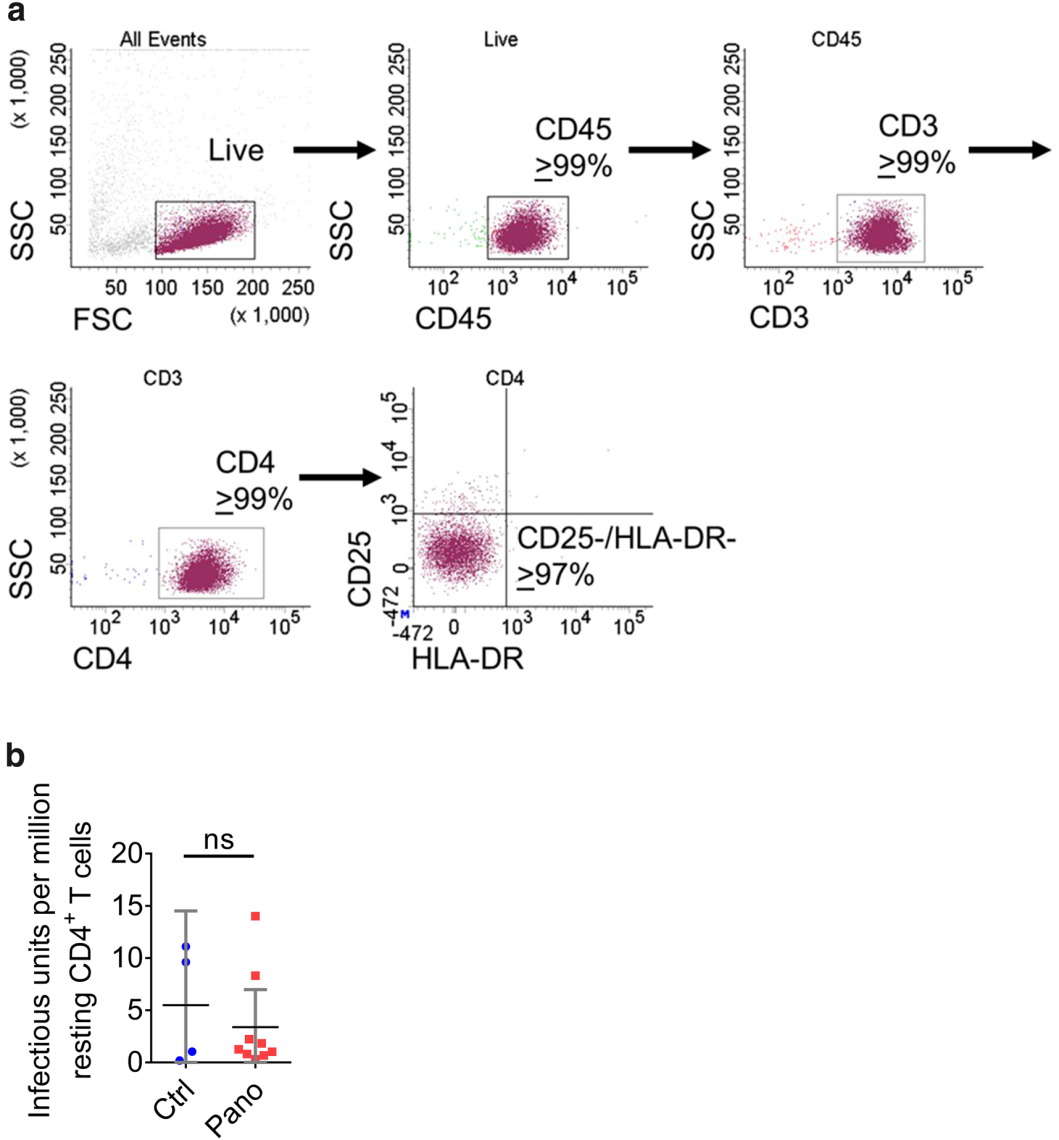
Analysis of panobinostat treatment on HIV latency in infected, suppressed BLT mice. BLT mice were infected with HIV-1_JR-CSF_, suppressed by antiretroviral therapy, and treated with panobinostat as described in Fig. 3. Resting human CD4^+^ T cells were isolated from the pooled tissues of each mouse, and analyzed by flow cytometry (**a**). Numbers of latently infected cells per million resting CD4^+^ T cells were determined by quantitative viral outgrowth assay. Median and interquartile range plotted with comparisons by Mann–Whitney test: ns p > 0.05. *Blue circles* denote control; *red squares* denote panobinostat-treated

In order to evaluate the effect of panobinostat on the latent HIV reservoir, we pooled cells from all the different tissues obtained from each HIV-infected ART-suppressed mouse and isolated resting human CD4^+^ T cells. The resting phenotype of the isolated cells was assessed by flow cytometry. Isolated resting cells were characterized by their lack of CD25 and HLA-DR cell surface expression (Fig. 6a).

Consistent with their resting state, the levels of HIV RNA in cells obtained from these well suppressed animals were below the limit of detection in two out of four samples from vehicle-treated mice; the levels of HIV RNA in the other two samples had an average of 512 copies per 100,000 resting CD4^+^ T cells. The levels of HIV RNA were below the limit of detection in eight out of nine samples from panobinostat-treated mice. In the single sample where we were able to quantitate HIV RNA in resting CD4^+^ T cells, it was 2100 copies per 100,000 resting cells. These low levels of HIV RNA in resting cells are consistent with results from human samples [49] and further illustrate the fact that, as we have previously published [41], in our QVOA analysis we are indeed evaluating latently infected resting cells.

To determine the levels of latently infected cells containing replication-competent proviruses, resting cells were first incubated with antiretroviral drugs overnight, then washed and plated for QVOA using PHA for maximal stimulation, essentially as we have previously described [41]. Under our experimental conditions, the levels of latently infected cells per million resting CD4^+^ T cells (IUPM) between the control and the panobinostat-treated groups were not statistically significantly different (p > 0.05) (Fig. 6b). Together, these results demonstrate that panobinostat administration at this dosing did not result in statistically significant differences in levels of cell-associated HIV RNA, HIV DNA, or latently infected cells in the tissues of HIV-infected, ART-suppressed BLT mice.

## Discussion

Even with effective antiretroviral therapies, a cure for HIV remains elusive, due to persistent replication in lymphoid tissue sanctuaries and latent infection of resting CD4^+^ T cells. Strategies to reverse latency and clear persistent infection may bring us closer to a cure by (1) inducing HIV expression in latently infected cells through latency-reversing agents, followed by (2) clearance of those cells mediated by viral cytopathic effects, immune clearance, or targeted cytotoxic agents. In this study, we evaluated the effect of histone deacetylase inhibitor panobinostat to modulate levels of cell-associated HIV RNA and HIV DNA as well as the size of the latent reservoir in BLT humanized mice. First, we observed that panobinostat treatment in vitro increased histone acetylation in uninfected human PBMCs and modestly induced HIV RNA expression in primary resting CD4^+^ T cells isolated from HIV-infected, ART-suppressed human subjects (Fig. 1). We also showed that ex vivo treatment with panobinostat induced outgrowth of replication-competent HIV from latently infected resting cells of several HIV-infected, ART-suppressed patients. Our results confirm and support previous results that panobinostat can reverse HIV latency in vitro [20, 24]. Next, panobinostat demonstrated histone deacetylase inhibition in BLT humanized mice: histone acetylation levels were significantly higher 24 h post-dose in tissues obtained from panobinostat-treated BLT mice compared to vehicle-treated animals (Fig. 2).

Following this demonstration of panobinostat bioactivity in BLT mice, we tested panobinostat in infected BLT mice suppressed with ART. We have previously shown that, in infected BLT mice, systemic levels of HIV RNA plateau 4 weeks after ART initiation [36]. In the current experiments, BLT mice received two additional weeks of ART to ensure that steady state levels had been reached. When we administered a 2-week course of panobinostat (four doses of 2 mg/kg, 3–4 days apart) to BLT mice that were infected with HIV and suppressed on ART, we did not observe a significant difference in levels of cell-associated HIV RNA between panobinostat-treated and vehicle-treated BLT mice, 4 days after the last panobinostat administration (Fig. 4). Given previous evidence that panobinostat activates transcription of HIV in vitro [20, 23, 24], we would have predicted an increase in cell-associated HIV RNA in panobinostat-treated BLT mice. However, we have shown that, while ART dramatically reduces levels of cell-associated HIV RNA in BLT mice, actively infected cells persist and continue to express HIV RNA [36]. If panobinostat did produce a specific effect of HIV activation in a rare population of latently infected cells, the increase in HIV transcription from these cells appeared to be unobservable in the context of persistent HIV RNA expression within tissues, at the timepoint they were harvested.

If latency-reversing agents induce HIV expression in latently infected cells, then infected cells could be cleared by viral cytopathic effects or immune mechanisms; and if this effect were sufficiently profound, a reduction in cell-associated HIV DNA might be measurable. However, we did not observe a significant difference in the levels of cell-associated HIV DNA at harvest between panobinostat-treated compared to vehicle-treated BLT mice (Fig. 5). This result may be explained by the predominance of persisting actively infected cells [36] or of cells infected with defective proviruses [3, 50, 51] that do not encode viral antigens to allow clearance, thereby masking the potential reduction in cells carrying HIV DNA. Also, the quantification of HIV DNA in this study did not include measurement of integrated HIV DNA specifically. Some investigators have suggested that integrated DNA might correlate more significantly with the frequency of latently infected resting CD4^+^ T cells [51]. The adaptation and validation of integrated HIV DNA quantification assays in fully suppressed BLT mice might be useful for an additional measure of the HIV reservoir in these models.

In order to more directly measure the frequency of cells containing latent, replication-competent HIV, we isolated resting human CD4^+^ T cells from the mice and utilized a QVOA, and a significant difference was not observed between the panobinostat- and vehicle-treated groups (Fig. 6). Based on the results presented in Fig. 1c showing approximately 500 infectious units per billion cells, the size of the latent reservoir in humanized mice might appear to be one order of magnitude greater than in humans. We should note that the amount of viral outgrowth from the human samples in Fig. 1c were measured after no treatment or after panobinostat treatment. These results do not reflect the total level of latent infection which would be measured after maximal stimulation by PHA treatment. The amount of viral outgrowth from the humanized mouse samples in Fig. 6b were measured after maximal stimulation by PHA treatment which likely explains the difference.

It should be noted that, while humanized mice are advantageous for studying HIV persistence in tissues, the limited amount of peripheral blood that can be collected makes it impractical to study the peripheral blood latent reservoir. Therefore, our data reflect tissue reservoirs, in contrast to the peripheral blood reservoirs typically measured in patients. Also, although we did not measure histone acetylation levels directly in BLT ART-suppressed mice, an increase in histone acetylation was demonstrated in uninfected mice in Fig. 2, and the ability of panobinostat to induce histone acetylation in humans undergoing ART has been previously demonstrated [24]. Notwithstanding, these results are similar to and complement those in previously reported clinical trial results of panobinostat in HIV patients [24]; and they could be explained by insufficient induction of latent proviral genomes, insufficient clearance of latently infected cells, or a combination of the two.

One important aspect of the kick and kill approach to eradication is the implementation of effective killing strategies. Recent evidence suggests that panobinostat might alter cytotoxic T lymphocyte activity. Some groups have published observations that HDAC inhibitors may enhance the effector responses of CD8^+^ T cells [52–55], while others have observed that HDAC inhibitors may reduce the ability of CD8^+^ T cells to clear HIV-infected cells [56]. It will be of great interest in future studies to investigate the functional ability of CD8^+^ T cells to clear HIV-1 infected cells in vivo and the possible confounding effect of latency-reversing agents.

Nevertheless, the lack of a quantifiable response to panobinostat in this model is consistent with the limited increases observed in vitro, specifically in the induction of HIV replication from latently infected resting CD4^+^ T cells as demonstrated in the QVOA utilizing highly relevant patient samples. It is possible that a modified dosing or sampling regimen may reveal efficacy in our system, and this will require further study. Targeted cytotoxic agents [36, 57, 58] may be necessary as well to enable clearance. Additionally, alternative HDACis, such as romidepsin [21, 59–62], or combinations of HDACis with other latency-reversing agents may further enhance HIV reactivation [23, 63] and should be tested in future experiments.

For example, NOD/*Rag1*^null^/*IL2Rgamma*^null^ mice injected intrahepatically with human liver-derived CD34^+^ cells (NRG-hu mice) have been used to test the effect of a combination of inducers—vorinostat, I-BET151, αCTLA4—and broadly neutralizing antibodies. A significant number (57 %) of mice treated with the combination of inducers and antibodies failed to rebound upon therapy interruption. Cell-associated HIV RNA and DNA were largely undetectable (11/13 and 5/13, respectively) in the spleens of non-rebounders, but the frequency of latently infected cells was not quantified [64].

The data presented herein demonstrate one clear parallel between what is observed in humans and BLT humanized mice. In humans, panobinostat treatment did not result in a measurable decrease in the levels of latently infected cells. Similarly, in BLT mice, we did not observe a difference in the levels of latently infected cells between panobinostat-treated and vehicle-treated animals. The fact that treatment with a single latency-reversing agent did not result in measurable reductions in the levels of latently infected cells in humans or BLT humanized mice serves to highlight the need for the evaluation of latency-reversing agent combinations. The implementation of therapeutic interventions that couple suppressive ART with combinations of latency-reversing agents requires a rational and comprehensive approach where the individual contribution of each agent alone is evaluated, and animal models that reflect the human condition will be useful for accelerating this progress towards an HIV cure [65].

## Conclusion

We have performed a systemic in vivo analysis in BLT humanized mice of the effect of panobinostat on histone acetylation and on cell-associated HIV RNA, DNA, and latent infection. After panobinostat treatment, we observed robust levels of systemic histone acetylation in BLT mice. However, we did not observe a statistically significant difference in the levels of cell-associated HIV RNA or HIV DNA in HIV-infected, ART-suppressed BLT mice. These results are consistent with the modest effects of panobinostat noted in vitro and in HIV-infected patients. Consistent with the results obtained in humans, in BLT mice panobinostat administration did not result in a decrease in the levels of latently infected resting CD4^+^ T cells. Together, the results obtained in both systems suggest that combination therapies may be necessary to reverse latency and enable clearance.
